# Intrinsic Cholinergic Neurons in the Hippocampus: Fact or Artifact?

**DOI:** 10.3389/fnsyn.2016.00006

**Published:** 2016-03-10

**Authors:** Jan Krzysztof Blusztajn, Jasmine Rinnofner

**Affiliations:** ^1^Department of Pathology and Laboratory Medicine, Boston University School of MedicineBoston, MA, USA; ^2^Department of Applied Life Sciences, University of Applied SciencesVienna, Austria

**Keywords:** acetylcholine, choline acetyltransferase ChAT, vesicular acetylcholine transporter, hippocampus, transgenic mouse models, basal forebrain cholinergic neurons, septum, slc18a3

## Abstract

It is generally agreed that hippocampal acetylcholine (ACh) is synthesized and released exclusively from the terminals of the long-axon afferents whose cell bodies reside in the medial septum and diagonal band. The search for intrinsic cholinergic neurons in the hippocampus has a long history; however evidence for the existence of these neurons has been inconsistent, with most investigators failing to detect them using *in situ* hybridization or immunohistochemical staining of the cholinergic markers, choline acetyltransferase (ChAT) or vesicular acetylcholine transporter (VAChT). Advances in the use of bacterial artificial chromosome (BAC) transgenic mice expressing a reporter protein under the control of the genomic elements of the *Chat* gene (*Chat*-BAC mice) have facilitated studies of cholinergic neurons. Such mice show robust and faithful expression of the reporter proteins in all known cholinergic cell populations. The availability of the *Chat*-BAC mice re-ignited interest in hippocampal cholinergic interneurons, because a small number of such reporter-expressing cells is frequently observed in the hippocampus of these mice. However, to date, attempts to confirm that these neurons co-express the endogenous cholinergic marker ChAT, or release ACh, have been unsuccessful. Without such confirmatory evidence it is best to conclude that there are no cholinergic neurons in the hippocampus. Similar considerations apply to other BAC transgenic lines, whose utility as a discovery tool for cell populations heretofore not known to express the genes of interest encoded by the BACs, must be validated by methods that detect expression of the endogenous genes.

The hippocampus continues to fascinate neuroscientists as a brain region essential for encoding and retrieval of memory [e.g., (Bird and Burgess, 2008; Augustinack et al., 2014)]. One of the key components of the neuronal circuitry necessary for these processes is the innervation of the hippocampus by basal forebrain cholinergic neurons (BFCN), which provides modulatory input mediated by the neurotransmitter, acetylcholine (ACh; Teles-Grilo Ruivo and Mellor, 2013). A decline in BFCN function and diminished cholinergic marker expression is apparent in aged humans and animals (Sarter and Bruno, 2004; Gauthier et al., 2006; Haense et al., 2012), in patients with Alzheimer’s disease (AD; Whitehouse et al., 1982; Bowen et al., 1983; Mufson et al., 2008; Grothe et al., 2012) and in animal models of AD (Savonenko et al., 2005; Payette et al., 2007; Perez et al., 2007; Goto et al., 2008; Mufson et al., 2008; Machová et al., 2010; Nikolajsen et al., 2011; Burke et al., 2013; Mellott et al., 2014). Thus, it has been postulated that abnormal cholinergic neurotransmission, due to dysfunction and/or degeneration of BFCN, contributes to the memory deficits seen in advanced age and in AD (Mufson et al., 2008; Grothe et al., 2012; Haense et al., 2012). Moreover, unlike the cerebral cortex, the hippocampus is characterized by a life-long capacity for neurogenesis that is essential for its normal function (Lee et al., 2012; Aimone et al., 2014), and age-related slowing of neurogenesis contributes to cognitive decline (Lee et al., 2012; Samson and Barnes, 2013; Aimone et al., 2014; Gray and Barnes, 2015). Studies in rats and mice indicate that cholinergic projections to the dentate gyrus promote adult hippocampal neurogenesis (Cooper-Kuhn et al., 2004; Mohapel et al., 2005; Van der Borght et al., 2005; Fontana et al., 2006; Kaneko et al., 2006; Kotani et al., 2006, 2008; Aztiria et al., 2007; Zhao et al., 2008; Fréchette et al., 2009; Ho et al., 2009; Narimatsu et al., 2009; Van Kampen and Eckman, 2010; Itou et al., 2011; Rennie et al., 2011). Therefore, investigations of cholinergic mechanisms in the hippocampus are motivated by both basic science- and translational objectives.

It is generally agreed that hippocampal ACh is produced and released exclusively by the terminals of BFCN. However, several studies performed in rodents have suggested the existence of a minor pool of intrinsic cholinergic neurons in the hippocampus. The support for this notion is based entirely on the immunohistochemical localization, within a small number of hippocampal cell bodies, of the enzyme choline acetyltransferase (ChAT) that catalyzes the synthesis of ACh and serves as the *sine qua non* marker of cholinergic neurons. As yet, no consensus on the existence of intrinsic hippocampal cholinergic neurons based on this method has been reached, because of studies that failed to show ChAT-positive neurons in the hippocampus (Armstrong et al., 1983; Ichikawa and Hirata, 1986), and because data on the distribution and morphology of the ChAT-positive hippocampal cells have been inconsistent (Clarke, 1985; Wainer et al., 1985; Frotscher et al., 1986; Matthews et al., 1987; Blaker et al., 1988; Kanaya-Ida and Ben Ari, 1989). Moreover, *in situ* hybridization failed to detect *Chat* mRNA-positive neurons in the hippocampus (Oh et al., 1992; Lauterborn et al., 1993). Once synthesized by ChAT, ACh is transported to the synaptic vesicles in a process catalyzed by the vesicular acetylcholine transporter (VAChT), encoded by the gene *Slc18a3*. The *Chat* and the *Slc18a3* genes co-localize within the genome, forming the cholinergic gene locus (Eiden, 1998; for certain *Chat* transcripts, *Slc18a3* may even be considered as one of their introns) and are co-expressed (Berse and Blusztajn, 1995). Therefore, VAChT constitutes another specific marker of cholinergic neurons. However, no studies that examined the immunohistochemical (Gilmor et al., 1996, 1998; Roghani et al., 1998; Schäfer et al., 1998) or *in situ* hybridization (Roghani et al., 1996; Ichikawa et al., 1997) localization of VAChT found cellular staining in the hippocampus. Likewise, direct comparison of *in situ* hybridization and immunohistochemical staining for ChAT and VAChT failed to detect positive cellular staining in the hippocampus (Ichikawa et al., 1997). Taken together, the available data based on immunohistochemical and *in situ* hybridization methods indicate that the presence of intrinsic cholinergic neurons in the hippocampus of mice and rats is unlikely.

In recent years, the availability of transgenic mice engineered for easy visualization of cholinergic neurons by the expression of indicator (e.g., fluorescent) proteins under the control of genomic elements surrounding the cholinergic gene locus, has generated a renewed interest in the intrinsic hippocampal cholinergic neurons because, in many cases hippocampal fluorescent protein-expressing cells have been observed. The general strategy for the construction of this type of transgenic mice, pioneered, perfected, and popularized by the GENSAT project (Gong et al., 2003, 2007; Heintz, 2004), relies on the use of bacterial artificial chromosomes (BACs) large enough to contain the entire cholinergic gene locus and surrounding genomic sequences thought to permit the locus expression exclusively in cholinergic cells. The BACs are first modified to encode a fluorescent protein (e.g., EGFP) or Cre recombinase, and are then used to generate transgenic mouse lines. Note, that depending on the BAC used, these types of transgenic mice may express additional genes that are present in the BAC together with the gene of interest (so-called “passenger” genes; Ting and Feng, 2014). Many of the *Chat*-BAC mice have the *Chat* gene disrupted by the fluorescent protein- or Cre-encoding gene but must be considered as transgenic for *Slc18a3*/VAChT (Tallini et al., 2006; von Engelhardt et al., 2007; Grybko et al., 2011; Zhao et al., 2011), unless this gene has been purposefully inactivated (Ting and Feng, 2014). The overexpression of VAChT in some of these mouse lines is reportedly associated with novel phenotypes (Nagy and Aubert, 2012, 2013, 2015; Kolisnyk et al., 2013; Crittenden et al., 2014). The *Chat*-BAC mouse lines show excellent and faithful transgene reporter expression in cholinergic cells, i.e., fluorescent proteins mark the appropriate neuronal populations (e.g., motor neurons, interneurons in the striatum, projection neurons of the medial habenula, and BFCN) and have the appropriate phenotypes including the expression of endogenous *Chat* as visualized by immunostaining. Thus, it is clear that studies of these neuronal populations provide information about the function and properties of *bona fide* cholinergic neurons. In our hands, one of these lines (B6.Cg-Tg(RP23-268L19-EGFP)2Mik/J; Tallini et al., 2006) has permitted studies on BFCN purified by fluorescence-activated cell sorting (Schnitzler et al., 2008, 2010) and on the effects of growth factors on BFCN in culture (Schnitzler et al., 2010) as well as *in vivo* in transgenic mouse models of AD (Burke et al., 2013; Mellott et al., 2014). Unfortunately, transgene reporter expression in *Chat*-BAC mouse lines is sometimes seen in brain regions not known for the presence of cholinergic neurons, such as the hippocampus. Figure 1 shows an example from this laboratory. We used the B6.Cg-Tg(RP23-268L19-EGFP)2Mik/J mice and examined ChAT-immunofluorescence and native EGFP fluorescence by confocal microscopy in brain sections. We found excellent colocalization of the EGFP and ChAT signals in the neurons and neuronal processes in septal BFCN. In contrast, the fluorescent EGFP-expressing cells in the hippocampus showed minimal, or no ChAT staining, and their processes were devoid of the ChAT signal even though most of the processes in this region showed colocalization of ChAT and EGFP, indicating that our immunostaining has adequate sensitivity for the detection of ChAT even in small structures. The latter fibers undoubtedly represent the septohippocampal afferents. In a report published recently, Yi et al. (2015) examined the electrophysiological and molecular phenotypes of fluorescent hippocampal cells using *Chat*-EGFP and *Chat*-Cre (crossed with Rosa26EYFP mice to generate EYFP-expressing cells) mouse lines; see also the associated editorial by Vijayaraghavan and Sharma (2015). They appropriately used quotation marks when referring to these cells as “cholinergic neurons” because while the cells exhibited the reporter protein expression, they did not stain for ChAT, indicating that they did not produce ACh. Moreover, the authors were unable to show ACh release from these cells. Instead these “cholinergic neurons” released glutamate upon depolarization and thus must be considered as glutamatergic. We conclude that the data reported by Yi et al. (2015), together with our observations (Figure 1), indicate that the adult hippocampus is devoid of intrinsic cholinergic neurons, consistent with the studies cited above. Whether such neurons occur early in development remains to be determined. If they do however, one would expect a relatively high number of EYFP-expressing hippocampal cells to arise from the Cre-mediated recombination in the *Chat*-Cre × Rosa26EYFP crosses performed by Yi et al. (2015). This, however was not observed, indicating that inappropriate activity of the *Chat-*BAC promoter constitutes a rare event in these mice, as acknowledged by Yi et al. (2015). The ectopic expression of the promoter may suggest that the BACs used for the generation of these mice are too short and do not contain all of the *cis*-DNA segments necessary for the appropriate silencing of the cholinergic gene locus in all non-cholinergic cells, or that the chromatin elements in the regions of BAC integration within the murine genome can occasionally override the normal control of the cholinergic gene locus. We note that the *Chat*-BAC mouse lines frequently harbor multiple copies of the BAC and that, to date, no studies on the integration sites of any of these BACs have been reported. Some of the drawbacks of the BAC-mediated transgenesis can be obviated by a knock-in strategy to produce comparable transgenic mice. Successful examples of this approach are lines in which a Cre allele, that is expressed via an internal ribosome entry site, was inserted downstream of the stop codon of the endogenous *Chat* gene as described by Rotolo et al. (2008) and Rossi et al. (2011). The former line has been used to investigate hippocampal cholinergic innervation. Consistent with the results of studies obtained with immunohistochemical methods, no intrinsic hippocampal cholinergic cells have been reported in these mice (Wu et al., 2014). In sum, the experience of the studies of *Chat*-BAC transgenic mice indicates that they are an excellent tool for the determination of the properties and physiological functions of *bona fide* cholinergic neurons in health and disease. However, their use to discover novel populations of cholinergic cells requires caution and rigor. If the only evidence for a new class of cholinergic neurons is the expression of a reporter protein in a *Chat*-BAC mouse line, it is best to conclude that such a class does not exist. Because there are now many BAC transgenic lines available to investigators, we posit that similar considerations should be applied to those.

**Figure 1:**
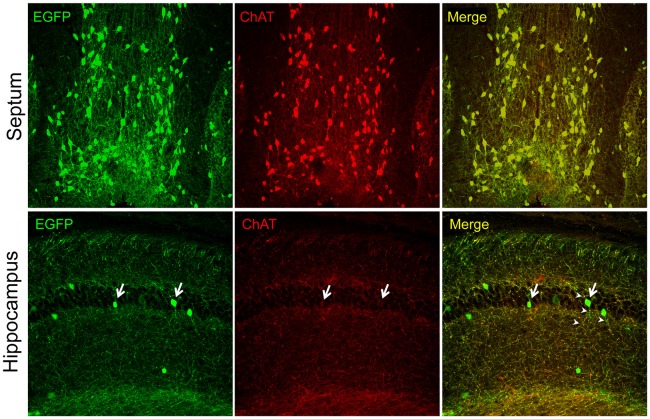
**Intrinsic EGFP fluorescence and choline acetyltransferase (ChAT) immunofluorescence (Millipore AB144P; 1:200) in brain sections of the 21 days old ChAT-EGFP mice visualized with confocal microscopy.** Note, strong ChAT staining and colocalization of the EGFP and ChAT signal (red) in the septal cell bodies and fibers (top panels). In contrast, the hippocampus (bottom panels) shows minimal (arrows) or no ChAT cell body staining and the colocalization of the EGFP and ChAT signals is seen only in fibers that do not emanate from the hippocampal cell bodies (presumably the septohippocampal afferents). The fibers of the EGFP-expressing cells do not show ChAT-immunostaining (arrow heads). Original magnifications: 10× (top panels) and 20× (lower panels). All procedures were performed in accordance with the protocol approved by the Boston University IACUC.

## Author Contributions

JKB conceived and wrote the manuscript. JR performed the study and wrote the manuscript.

## Funding

Supported by an NIH grant AG045031. JR was supported by the Austrian Marshall Plan Foundation.

## Conflict of Interest Statement

The authors declare that the research was conducted in the absence of any commercial or financial relationships that could be construed as a potential conflict of interest.
